# Comparison of sample types from white-tailed deer (*Odocoileus virginianus*) for DNA extraction and analyses

**DOI:** 10.1038/s41598-021-89390-2

**Published:** 2021-05-11

**Authors:** Jessie Edson, Justin Brown, William L. Miller, W. David Walter

**Affiliations:** 1 Pennsylvania Cooperative Fish and Wildlife Research Unit, The Pennsylvania State University, 413 Forest Resources Building, University Park, PA 16802 USA; 2 Department of Veterinary and Biomedical Sciences, The Pennsylvania State University, University Park, PA 16802 USA; 3 Department of Biology, Calvin University, 1726 Knollcrest Circle SE, Grand Rapids, MI 49546 USA; 4 U.S. Geological Survey, Pennsylvania Cooperative Fish and Wildlife Research Unit, The Pennsylvania State University, 403 Forest Resources Building, University Park, PA 16802 USA

**Keywords:** Ecological epidemiology, DNA sequencing

## Abstract

Collection of biological samples for DNA is necessary in a variety of disciplines including disease epidemiology, landscape genetics, and forensics. Quantity and quality of DNA varies depending on the method of collection or media available for collection (e.g., blood, tissue, fecal). Blood is the most common sample collected in vials or on Whatman Flinders Technology Associates (FTA) cards with short- and long-term storage providing adequate DNA for study objectives. The focus of this study was to determine if biological samples stored on Whatman FTA Elute cards were a reasonable alternative to traditional DNA sample collection, storage, and extraction. Tissue, nasal swabs, and ocular fluid were collected from white-tailed deer (*Odocoileus virginianus*). Tissue samples and nasal swabs acted as a control to compare extraction and DNA suitability for microsatellite analysis for nasal swabs and ocular fluid extracted from FTA Elute cards. We determined that FTA Elute cards improved the extraction time and storage of samples and that nasal swabs and ocular fluid containing pigmented fluid were reasonable alternatives to traditional tissue DNA extractions.

## Introduction

Genetic testing and monitoring have become common components of wildlife research. Genetic techniques, such as next-generation DNA sequencing^1^, DNA barcoding^1,2^, and genetic fingerprinting^3,4^, and quantitative polymerase chain reaction (qPCR)^5^ are now being applied to the study of non-model organisms. These analyses provide important information regarding phylogenetic history^6,7^, genetic structure^8,9^, risk of disease^10^, and viability of wildlife populations^11,12^. These analyses are also used for forensic studies and can aid law enforcement efforts^2,13,14^.

The success of genetic studies is dependent upon genetic sampling which involves capture and extraction of mitochondrial or nuclear DNA from cells of the target organism^15^. Various quantities of DNA can be isolated from a large array of potential sources including connective and integumentary tissues, blood, hair, feathers, feces, saliva, urine and bone^16^. Freezing, drying and the use of additives help to prevent cellular breakdown and preserve genetic samples until DNA extraction can be performed^16^. Many high-quality samples, such as tissue and blood, require immediate freezing or preservatives to minimize damage to DNA, which can add challenges for field collection^16^. Determining the most appropriate source of DNA to collect depends on the field conditions, availability of sample types, shipping and storage requirements, and the research objectives.

White-tailed deer (*Odocoileus virginianus*) are a widely studied and managed species due to their popularity amongst hunters and their role in shaping community structure and diversity of ecosystems where they occur^17^. Concerns such as the spread of chronic wasting disease documents a continued need for genomic research of white-tailed deer^18^. Wildlife researchers and managers commonly retrieve tissue samples from deer collected through roadkill, hunter harvest and tagging efforts^8,19^. Blood is another commonly obtained sample^20,21^, and some research has utilized deer feces for genotyping studies^22,23^. Although samples are readily available from a variety of sources, DNA degradation, long-term storage, contamination, and transportation of samples continue to be potential challenges to genotyping efforts. Traditional methods for blood and tissue collection have disadvantages, such as the training needed to extract blood and the need for anticoagulants/reagents for storing and shipping samples. Other issues arise when the most practical sampling, due to cost and availability, relies on cooperation with another research project, such as tagging or disease surveillance, where DNA sampling may not be the primary concern of technicians. In some of these instance’s samples are collected in large quantities and grouped together and contamination from other specimens is a possibility to outer surfaces.

An alternative for DNA collection and storage that circumvents many challenges is the Whatman Flinders Technology Associates® Card (hereby referred to as FTA cards; QIAGEN, Valencia, CA, USA). These FTA cards are filter paper infused with reagents that lyse cells and denature protein, in addition to protecting DNA from UV radiation, nucleases, oxidation and microbial or fungal damage^24^. FTA cards are designed for compact storage of DNA samples at ambient temperature, which may be particularly beneficial for field-based research^25^. Another advantage is that they can be shipped in standard mail, since they need no additional reagents and are stable at room temperature, they require no special transport formats^24^. FTA cards have been used in a large variety of studies including: SNP genotyping in angus bulls where blood and nasal swabs were compared^26^, cancer screening in humans using buccal swabs^27^, amplification in PCRs from a variety of wildlife utilizing blood and tissue^28^ and assessing *Borrelia* spp. bacteria in white-tailed deer and canids that had been potentially infected by ticks using mitochondrial DNA from blood samples^29^. FTA cards have been used in remote field expeditions, utilizing a variety of sources such as feces, buccal cells, and blood samples^25^. These studies highlight the potential value and utility of FTA cards for wildlife genetic studies. Use of FTA cards would enable hunters, managers, and researchers to sample white-tailed deer in the field reducing the need for large collection locations and movement of carcasses to central sampling locations due to disease concerns^18^. Additionally, FTA cards denature infectious microorganisms which makes them an appealing option when working with samples where concern about diseased animals may be an issue^30^.

The purpose of our study was to examine alternative DNA sources that could be stored and extracted using FTA cards while reducing the difficulty of sampling and decreasing the risk of contamination. We specifically focused on evaluating nasal swabs as a potential alternative because previous research suggested that nasal swabs would be suitable for use on live deer^31^ and may be less invasive and less training intensive than traditional sampling methods. We assessed ocular fluid, as we assumed it would be less likely to have surface contaminates, since we could draw fluid from within the eye. We assumed collecting ocular fluid would result in less errors than extracting blood from a carcass, where the blood may clot depending on the length of time between death and when sample collection occurs.

## Methods

We selected Whatman FTA Elute Micro non-indicating cards (referred to as FTA Elute cards) (QIAGEN, Valencia, CA, USA), since they contain a similar chemical makeup to the classic cards and serve the same functions but are designed to have a simplified DNA elution step and a higher DNA yield than traditional cards^24^. For ocular fluid, indicator cards could have been advantageous, because they change color when a liquid is added, and the fluid was sometimes clear with no visible pigment.

Genetic samples were collected from 38 free-ranging white-tailed deer using various strata (e.g. hunter harvest, roadkill surveillance, and targeted removal) during sampling for chronic wasting disease in Pennsylvania, USA. All deer were sampled by the same veterinarian within 72 h of death. Tissue samples were collected as ear biopsies from all 38 deer and kept frozen at − 20 °C until DNA extraction was performed. From a subset of the 38 deer, nasal swabs (n = 22) and/or ocular fluid (n = 27) were collected and inoculated onto FTA Elute cards based on manufacturer’s instructions. Nasal swabs were collected by inserting a sterile cotton-tipped applicator (Puritan, Hardwood Products Company, Guildford, Minnesota, USA) into the nasal cavity and immediately rolling it onto the FTA Elute card. Ocular fluid was collected by inserting a 22-gauge needle into the anterior chamber and directing it toward the angle of the eye and approximately 40 μl of fluid was added to the FTA Elute cards. Of the 27 ocular fluid samples, 13 were clear and 14 contained grossly visible flecks of dark brown pigment. The FTA Elute cards were air dried for 24 h at room temperature and then stored frozen at − 20 °C until samples were extracted. FTA cards have been reported to work stored at room temperature or frozen at − 20 °C or − 80 °C^32^ and freezing is suggested by the manufacturer for RNA samples on FTA cards, which are less stable than DNA samples. Since samples were stored frozen prior to obtaining them in the lab we chose to keep all FTA Elute card samples frozen. Nasal swabs were placed in a sterile, empty vacutainer tube (Becton, Dickinson and Company, Franklin Lakes, NJ, USA) after FTA Elute card inoculation and frozen at − 20 °C until direct extraction was performed.

Tissue samples and nasal swabs were extracted using QIAGEN DNeasy Tissue and Blood Extraction Kit (QIAGEN, Valencia, CA, USA). The QIAGEN DNeasy tissue extraction protocol was followed, with one minor modification of eluting with 150 µL AE buffer, instead of 200 µL AE buffer at the final step. Tissue samples underwent a 4 h to overnight digestion in the proteinase K solution, which breaks down proteins. For nasal swabs the QIAGEN DNeasy tissue extraction protocol was also followed by using approximately 1/5th of the cotton swab and digesting it for 3 or more hours in the proteinase K solution at 56 °C. The samples were then centrifuged with subsequent removal of the cotton prior to following the remaining steps of the tissue extraction protocol.

Samples stored on FTA Elute cards were extracted following the manufacturer's protocol, except we utilized an approximate 4 mm square of sample to increase the amount of retrievable DNA, instead of the recommended 3 mm punch. We reextracted 20 samples stored on FTA cards 16–21 months after they were collected utilizing the same techniques. FTA cards were extracted separately and at a different time then tissue and nasal swab samples to minimize the risk of contamination between sample types. All sample types were cut out on a new piece of parafilm with a new razor blade and handled with tweezers that were flame sterilized in between each sample to prevent any contamination. A NanoDrop spectrometer (ThermoFisher Scientific, Waltham, MA, USA) was used to determine the quantity and quality of 1 µL eluted DNA for each sample (Table 1).

**Table 1:** Results of DNA quantification by nanodrop spectrometer for different DNA sample types: tissue, nasal swab, nasal swab on an FTA Elute card, pigmented ocular fluid on an FTA Elute card and non-pigmented ocular fluid on an FTA Elute card. Also, results for samples that were reextracted 16–21 months after initial collection for pigmented ocular fluid on an FTA Elute card and nasal swab on an FTA Elute card. Results include the minimum, maximum, average and median value of DNA concentration for each sample type.

Sample type	Number of samples	DNA concentration Min (ng/µL)	DNA concentration Max (ng/µL)	Average (ng/µL)	Median (ng/µL)
Tissue	38	3.79	259.09	103.59	98.83
Ocular Fluid—pigmented	14	8.89	111.58	45.62	35.00
Ocular Fluid—non-pigmented	13	4.68	49.08	19.2	11.58
Nasal Swab	20	11.72	274.42	72.25	39.39
Nasal Swab-FTA	22	22.5	139.55	65.25	61.66
**Samples Reextracted after 16–21 months in storage**					
Nasal Swab—FTA	10	12.01	101.52	46.81	40.4
Ocular Fluid—pigmented	10	3.75	27.86	10.13	8.82

Samples were genotyped using a core 11-microsatellite panel developed for genotyping white-tailed deer^19^. Multiplex mixes were redesigned from previous research in order to be used effectively with FTA Elute cards^19^. We used QIAGEN Multiplex PCR Kit (QIAGEN, Valencia, CA, USA) to perform PCRs with 10 µL reaction volumes consisting of: 5 µL 2 × QIAGEN Multiplex PCR Master Mix, 1 µL Q_solution, 1 µL of 20 ng DNA and 3 µL of primer mixtures diluted to 20 µM in PCR grade water^19^. PCR conditions mostly followed recommendations from the QIAGEN Multiplex PCR kit, beginning with incubation at 95 °C for 15 min to activate the HotStarTaq DNA Polymerase (QIAGEN), followed by 32 cycles of denaturing at 94 °C for 30 s, annealing for 90 s at multiplex specific temperature (Table 2), and extension at 72 °C for 60 s, with a final extension step of 72 °C for 10 min. Multiplex arrangements can be found in Table 2. We mixed 1 µL of each PCR amplicon with 10 µL of a denaturing agent (Hi-Di Formamide, ThermoFisher Scientific, Waltham, MA, USA). Fragment size determination was carried out using an Applied Biosystems genetic analyzer (model 3730 XL; Waltham, MA, USA) at the Penn State Genomics Core Facility (University Park, PA, USA). We used the software GeneMarker (Softgenetics, State College, PA, USA) to determine allele identity by comparing the electrophoretic mobility of PCR amplicons to that of a known size standard (GeneScan™ LIZ™ Dye Size Standard; ThermoFisher Scientific, Waltham, MA, USA). A previously confirmed sample was added to each genotyping plate to standardize across runs. A no-template control sample was also included to check for contamination.

**Table 2:** Microsatellite multiplex combinations with primers grouped by multiplex with Motif = expected repeat motifs, Vol = volume of primer mix (µL), Dye = dye color, and AT = annealing temperature (°C). Multiplex panels and reaction conditions were adapted from Miller et al. (2019).

Locus	Primer sequence	Motif	Vol	Dye	AT	Range
**Multiplex A**						
RT9	F: TGAAGTTTAATTTCCACTCT R: CAGTCACTTTCATCCCACAT	2	0.20	6-FAM	57.0	102–125
BM4107	F: AGCCCCTGCTATTGTGTGAG R: ATAGGCTTTGCATTGTTCAGG	2	0.18	6-FAM	57.0	134–168
P	F: TTTCACTGTTTTCTCCTTCAGA R: TGCCCAATCAGATGTTGTAG	4	0.17	NED	57.0	210–244
Q	F: AATGTGTCAGTGAAGGTCTTC R: ATCCAGGCAACCATCTAG	4	0.18	6-FAM	57.0	228–295
**Multiplex B**						
RT5	F: CAGCATAATTCTGACAAGTG R: GTTGAGGGGACTCGACTG	2	0.15	6-FAM	60.0	98–125
BM6438	F: TTGAGCACAGACACAGACTGG R: ACTGAATGCCTCCTTTGTGC	2	0.13	NED	60.0	251–280
Cervid1	F: AAATGACAACCCGCTCCAGTATC R: TCCGTGCATCTCAACATGAGTTAG	2	0.10	NED	60.0	159–198
**Multiplex C**						
RT7	F: CCTGTTCTACTCTTCTTCTC R: ACTTTTCACGGGCACTGGTT	2	0.14	VIC	54.0	205–243
BL42	F: ACAAGTCAAGGTCAAGTCCAAATGCC R: CGATTTTTGTGTTAATTTCATGC	2	0.22	PET	54.0	235–266
INRA011	F: CGAGTTTCTTTCCTCGTGGTAGGC R: GCTCGGCACATCTTCCTTAGCAAC	2	0.17	PET	54.0	189–207
OarFCB193	F: TTCATCTCAGACTGGGATTCAGA R: GCTTGGAAATAACCCTCCTGC	2	0.16	NED	54.0	94–127

We evaluated the effectiveness of alternative methods of DNA collection by comparing PCR results to tissue controls. We classified results from microsatellite analysis to be successful if they amplified at a minimum of nine loci. Samples where alleles were not obvious or were difficult to interpret for more than two loci were rerun. Samples were classified as poor if they produced similar results for reanalyzed loci but had interpretable results at six to eight loci. Samples were classified as failed if we could not confirm allele calls for at least six loci following reruns. Samples considered successful and originating from the same host were then compared against each other and evaluated for mismatched alleles or amplification failure.

## Results

Each of the 38 deer sampled had an ear tissue biopsy and at least one other source of DNA for comparison. We determined quantity of DNA from the different sample types using a NanoDrop spectrometer and found that tissue samples had the highest average quantity, followed by nasal swabs, then nasal swabs on FTA cards, then pigmented ocular fluid, and non-pigmented ocular fluid had the lowest average (Table 1).

We calculated the amplification success rate, defined as the proportion of successful results that matched tissue controls, for each method (Table 3). Ocular fluid with pigment had the highest rate of success among the samples tested with a 100% success rate for 14 samples, while clear ocular fluid had the lowest success rate of only 15.4% for 13 samples (Table 3). Nasal swabs were 95% successful for both methods of extraction and ear tissue was 89.5% successful (Table 3).

**Table 3:** Results of DNA extraction for genetic samples collected from white-tailed deer in Pennsylvania, USA, for QIAGEN DNeasy and FTA card extraction protocols. The rate of successful amplifications (% Success) was determined for each collection method based on the number of samples with reliable PCR amplification for 11 microsatellite loci.

	Total samples	Successful amplification	Poor/failed amplification	% Success
QIAGEN Extraction Protocol				
Tissue	38	34	4	89.5%
Nasal Swab	20	19	1	95.0%
FTA Card Extraction Protocol				
Nasal Swab—FTA	22	21	1	95.5%
Ocular Fluid—Non-Pigmented	13	2	11	15.4%
Pigmented—Ocular Fluid	14	14	0	100.0%
**Reextraction of FTA Card Extraction Protocol after 16–21 months in storage**				
Nasal Swab—FTA	10	10	0	100.0%
Ocular Fluid—Pigmented	10	9	1	90.0%

We were able to obtain a full genotype for every individual, but not from every source of DNA. One nasal swab, and one nasal swab on the FTA card, produced poor results but were not from the same individual. Two of the ear tissue samples produced poor results and two failed, but the matching FTA card samples provided full genotypes. Of the 27 ocular fluid samples there were 13 non-pigmented FTA cards with five failed and six producing poor results, while all 14 pigmented FTA cards produced successful results.

We calculated error rates for successful results by examining them for mismatched allele calls and allele failures (Table 4). We had 1 nasal swab sample with a mismatched allele creating an error rate of 5.26%. We had 1 tissue sample fail to amplify an allele, producing an error rate of 2.94%. We had 2 pigmented ocular fluid samples fail to amplify an allele creating an error rate of 14.28%. We had 1 nasal swab FTA card fail to amplify an allele creating an error rate of 4.76%. We had 1 non-pigmented ocular fluid sample fail to amplify an allele creating an error rate of 50%.

**Table 4:** Error rate calculations for successful results determined by mismatched allele calls and failed allele calls.

Sample type	Mismatched Allele	Failed Allele	Number successful	Error rate
Tissue	0	1	34	2.94%
Ocular Fluid—Pigmented	0	2	14	14.28%
Ocular Fluid—non-pigmented	0	1	2	50%
Nasal Swab—FTA	0	1	21	4.76%
Nasal Swab	1	0	19	5.26%
**Samples Reextracted after 16–21 months in storage**				
Nasal Swab—FTA	1	0	10	10%
Ocular Fluid—Pigmented	0	1	9	11.11%

## Discussion

Ear tissue is a frequent DNA source for wildlife studies, having been used in studies on fisher (*Martes pennanti*)^33^, red fox (*Vulpes vulpes*)^34^, squirrel glider (*Petaurus norfolcensis*)^35^, snowshoe hare (*Lepus americanus*)^36^ and white-tailed deer^19,37^. While ear tissues for this study generally displayed high-quality results, some errors were observed. Ear tissue with poor results in our study were largely composed of cartilage without overlying epidemis, and did not digest well in our proteinase K solution. These samples were extracted a second time to rule out errors in the DNA extraction process and produced the same results. These samples were primarily cartilage, and tissue digestion using collagenase type II instead of proteinase K are more likely to be successful in such situations^38,39^. Nasal swabs were a viable source of DNA, producing adequate DNA concentration (Table 1), and having a success rate of 95% (Table 3). The DNA extractions from nasal swabs were performed using the same reagents and techniques as our tissue samples, and required about the same amount of time, but nasal swabs rolled on FTA cards improved the extraction time with no loss of genotyping efficiency (Table 3). Nasal swabs needed to be frozen and stored in a 10 ml vial, while FTA cards required less storage space and could either be stored frozen or at room temperature. The ocular fluid samples could be a valuable resource if pigmented cells are obtained when sampling (Table 3). Cytologically, the pigmented fluid consisted of numerous cells containing intracytoplasmic pigment (presumably pigmented epithelial cells of the iris)^40^. The clear ocular fluid samples were likely from the aqueous humor, the fluid that fills the anterior and posterior chambers of the eye, which contains few cells^40^, and would likely explain the poor performance of these sample types. Clear ocular fluid was classified as low-quality and could be useful when other samples are not available, but pigmented ocular fluid, nasal swabs and ear samples all provided good quantity and quality results and could be valuable resources for wildlife studies.

Ear tissue can be retrieved from live animals usually during wildlife capture efforts or from deceased animals. A concern with using ear samples is obtaining only cartilage, when it is best to also retrieve skin and connective tissue. Concerns for collection occurs when the ear becomes degraded when exposed to UV light and is more likely for the surface to become contaminated with blood and decay from other deer when carcasses are stored together for sampling. Furthermore, allelic dropout in red fox was reported using ear tissue^34^. Our results showed that both nasal swabs and pigmented ocular fluid provided a comparable or higher success rate for genotyping than ear tissue. Nasal swabs and ocular fluid both worked on FTA cards and could be useful sample types when there are concerns about contamination to outer surfaces from deceased deer. These concerns may arise when carcasses are commonly collected in large quantities at a central public repository for hunters, or at meat processors and taxidermists. The eye and nasal cavity would be less likely to become contaminated from bodily fluids in this instance. Likewise, with roadkill deer if the sample is not fresh, the nasal cavity may be a good option to sample from, because those cells are better protected from degradation by ultraviolet radiation^14^. Our study readily got good quality samples from deer that were deceased for up to 3 days before sampling. Also, while nasal swabs for this project were collected from deceased deer, there could be value in using nasal swabs on live specimens if less invasive sampling techniques are preferred over blood draws, ear clips, or muscle biopsies. Nasal swabs would require less training for personnel collecting the sample, since no sharps or technical skills like blood draws would be required.

FTA cards could provide wildlife researchers with a nice alternative to traditional sample collection, storage, and extraction. The FTA elute cards would be less expensive because fewer supplies were needed for sample collection, no extraction kit was required, and fewer technician hours were needed for extractions. The storage space requirements were dramatically improved, requiring only small plastic bags and less freezer space. The FTA cards can also be stored at room temperature^28^, further indicating their value when storage space is limited or when refrigeration is not available. There is a decreased risk of contamination when extracting DNA from FTA cards as fewer transfer steps are required, which also decreases the number of hours needed for DNA extraction, compared to the Qiagen kit method. A reduced heating step for the FTA cards over traditional methods (30 min heat step vs. 3 + hrs heat step) further reduces the time needed. Since there are no required preservatives, freezing, or bulky storage tubes needed, FTA cards could be beneficial for field crews who must carry in supplies. FTA cards may have additional added bonuses to wildlife studies as they can also be used for storing and extracting RNA samples^30^ and can be used for direct PCR amplification^41^. One draw-back to using FTA cards is that the extraction yield is significantly lower volume than the Qiagen kit extraction method (30µL vs. 200µL), which could lead to the need for duplicate extractions. Sufficient amount of sample is readily obtained, however, if duplicate extractions are performed. If the sample area, which is an 11 mm disk, is fully covered, then roughly eight extractions can be completed if using the 4 mm square size, which is what we used in this study.

We re-extracted 10 pigmented ocular fluid and 10 nasal swabs from FTA cards after being stored frozen at − 20 °C for 16–21 months and checked the DNA quantity using a nanodrop spectrometer. We compared those results to the original extractions and in both cases, we generally saw a decrease in DNA quantity (Table 1). However, when reevaluating the samples for genotyping across 10 of the 11 original microsatellites, all samples produced results. All reextracted samples would classify as successful*,* with the exception of one pigmented ocular fluid sample that failed at 2 markers. Despite the reduction in quantity, the genotyping results indicate that samples can still be used for genetic studies after extended storage. There was a greater decrease in the pigmented ocular fluid samples then in nasal swabs, which could warrant further studies on the longevity of these sample types (Table 1).

Another consideration for wildlife studies is non-invasive sampling. Many studies have used non-invasive sampling techniques, utilizing feces^23,42,43^ and hair samples^44,45^. Deer feces were used to genotype Sitka black-tailed deer to develop a population estimate based on unique genotypes Brinkman et al.^23^. After modifications to their collection and extraction procedures they had a genotyping success rate of 87%^23^. In a study comparing fecal samples to blood samples in bison, allelic dropout was found to be a significant source of error, accounting for 60.3% of mismatches in the study^42^. However, they were still able to select 15 microsatellites that matched 95% of the time to be able to generate unique and accurate genotypes^42^. Little research has been done on the efficiency of using FTA cards with non-invasive sample types and warrants further research.

Our comparison of different DNA sources and storage/extraction methods has shown that FTA cards could provide scientists with a reliable method of obtaining, storing, and extracting DNA, in comparison to traditional methods. The FTA Elute cards provide ease of storage, and have a simple, fast extraction process, requiring only the use of PCR grade water^24^. While our study focused on white-tailed deer samples, our research and previous research documented that FTA cards would be appropriate with other species^25,28^. Future research on the use of FTA cards with low quality samples would be beneficial for field collections using non-invasive genetic sampling.
